# Prevalence and predictors of depression and anxiety among medical students in Addis Ababa, Ethiopia

**DOI:** 10.1186/s13033-019-0287-6

**Published:** 2019-05-06

**Authors:** Mebratu Abraha Kebede, Birke Anbessie, Getinet Ayano

**Affiliations:** 1 Department of Research, Saint Paul Hospital Millennium Medical College, POBox 171, Addis Ababa, Ethiopia; 2 Research and Training Department, Amanuel Mental Specialized Hospital, Addis Ababa, Ethiopia

**Keywords:** Depression, Anxiety, Medical students, Predictors, Ethiopia

## Abstract

**Background:**

Depression and anxiety are among the common mental health problems among medical students and are associated with poor academic performance, disability and poor quality of life. A better understanding of the magnitude and correlates of depression and anxiety is essential for planning appropriate intervention for those population groups. However, research into depression and anxiety and the potential contributing factors is limited in low and middle-income countries including Ethiopia. Therefore, this study aimed to determine the prevalence and associated factors of depression and anxiety among medical students.

**Methods:**

A cross-sectional study was conducted among 273 medical students selected by systematic sampling technique. Hospital anxiety and depression scale (HADS) was used to assess anxiety and depression. Binary and multivariable logistic regression models were fitted, adjusting for the potential confounding factors. Odds ratios (OR) with the corresponding 95% confidence interval (95% CI) was computed to assess the strength of association.

**Result:**

The prevalence of co-morbid depression and anxiety was found to be 21.20% (16.35% to 26.05%) and prevalence of depression and anxiety was 51.30% (45.37% to 57.23%) and (30.1% 24.66% to 35.54%), respectively. Multivariate analysis showed that being female [AOR 2.56, 95% CI (1.32, 4.95)], first-year educational level [AOR 12.06, 95% CI (2.18, 66.72)], second-year educational level [AOR 8.99, 95% CI (1.67, 48.45)] and those who had poor/low social support [AOR 5.36, 95% CI (2.08, 13.76)] were significantly association with anxiety. Students who were in the age interval of 18–21 years [AOR 2.42, 95% CI (1.64, 9.22)], first-year educational level [AOR 1.63, 95% CI (1.43, 6.26)], second-year educational level [AOR 1.39, 95% CI (1.17, 5.18)] and who had stressful life events [AOR 1.61, 95% CI (1.14, 2.76)] were significantly associated with depression among medical students.

**Conclusion:**

The current study demonstrated that a remarkable proportion of medical students are suffering from depression (51.30%), anxiety (30.10%) as well as comorbid depression and anxiety (21.20%). There was strong evidence of association between anxiety and female sex, first-year educational level, second-year educational level and having poor/low social support. Whereas a significant association was observed between depression and younger age (18–21 years old), first-year educational level, second-year educational level and having one or more stressful life events in the last 6 months. Screening of depression and anxiety among medical students was recommended. Future studies focusing on better ways of preventing and treating depression and anxiety among medical students are warranted.

## Background

Depression is a significant public health problem and is characterized by sadness, loss of interest in activities and by decreased energy. It is differentiated from normal mood changes by the extent of its severity, the symptoms and the duration of the disorder [1]. Depression is the fourth most important contributor to the global burden of disease and 4.4% of the total disability adjusted life years (DAILY) is explained by depression [2, 3]. Epidemiologic evidence also showed that about 1.2% of the total burden in Africa to 8.9% in high-income countries is explained by depression [4].

Depression is common in university students especially it is high among medical students with no preponderance between males and females and in single students is higher than married ones [5, 6]. It may be a significant hidden problem in medical students and mechanisms to identify and help students with mental health problems should be seriously considered [7]. The high prevalence suggests that immediate preventive measures should be implemented, such as the setting up of psycho-pedagogic support services for students, and teacher development programs [8].

Anxiety is a vague feeling of apprehension, worry, uneasiness, or dread, the source of which is often non-specific or unknown to the individual [9]. Anxiety and depression have a huge effect on society and individual, which can lead to the suicidal tendency, relationship problems, medical dropouts, and impaired work ability. Therefore, proper counseling services required to the psychological well-being of medical students to improve their quality of life [10]. Symptoms of depression and anxiety disorders have the most significant impact on health-related quality of life (HRQOL) [11].

Psychological illnesses in the form of depression, anxiety, and stress have been reported in a substantial proportion of first-year medical students. Multiple social, demographic, behavioral, and academic factors have been found to be significantly associated with most of the studied psychological morbidities; among them, gender, residence, feeling loneliness, the inability to share families in social activities, presence of insomnia and chronic physical illnesses, studying in English language, problems with exams’ criteria, and the organization of lectures’ timetable were the most common [12].

Emotional disturbances in the form of depression and anxiety exist in high rate among undergraduate science students that require early intervention. Factors including the feeling of incompetence, lack of motivation to learn and difficulty of class work can be considered as a source of stressors that may precipitate for depression and anxiety [13].

Attending university is a particularly stressful time due to unique emergent stressors such as changes in environment, loss or diminishment of social support networks, academic pressures, developing peer relationships, and financial management [14]. The current educational process may have a negative effect on students’ mental health, with a high frequency of anxiety among medical students [15].

Even though depression and anxiety are found to be remarkably high among medical students coupled with their impacts in causing poor academic performance, disability and poor quality of life, to our knowledge a few studies are available in East Africa including the study area (Ethiopia). Therefore, this study aimed to assess the prevalence and factors associated with depression and anxiety among medical students in the study area.

## Methods and materials

### Study setting, study design and period

Institutional based cross-sectional study was conducted from April to May 2017, at St. Paul Hospital millennium medical college, Addis Ababa, Ethiopia.

### Study population

The study population consisted of All St. Paul Hospital millennium medical college medical students (from year one to internship) who were included in the sample.

### Sampling procedure

The sample size was determined based on a single population proportion formula using Epi-info version 7 with a 95% CI, 5% margin of error and taking the prevalence of depression and anxiety 27.7% [26] and 40.9% [25] respectively. By considering a 10% non-response rate and applying sample correction formula a total sample size of 273 undergraduate medical students were involved in the study.

We used a systematic random sampling technique to select two hundred seventy-three (273) medical students who were included in the study. We determined the sampling interval by dividing the total study population (medical students) by the total sample size which was two. The first study participant was selected using lottery method and the next study participants were choose at a regular interval (every second interval) and interviewed by data collectors.

### Data collection procedures and instrument

Data were collected using pretested an interviewer-administered questionnaire, which contains data on the outcome of interest (depression and anxiety), socio-demographic characteristics (age, sex, source of income and marital status), academic-related factors (academic year of study, duration of the vacation, impact of educational vocation and academic interest), clinical factors (family history of mental illness, chronic illness), psycho-social factors (social support, stressful life events) as well as substance-related factors (khat, alcohol, tobacco and others).

The hospital anxiety and depression scale (HADS) was used to measure depression and anxiety among medical students [16]. HADS was validated and extensively used in Ethiopia [17, 18]. The tool containing 14 questions with a cut-off score for depression and anxiety of greater than or equal to 8 [16].

Social support was measured by Oslo 3-item social support scale and individual who scored 3–8, 9–11, and 12–14 were considered as having poor, moderate and strong social support respectively [19].

Stressful life events were measured using self-report of experiencing one or more major stressful life events (including health-related problems, death significant others, and financial crisis) in the last 6 months.

In the current study, substance use such as alcohol, cigarette, and khat indicates current use. Of those substances. Those participants who have a history of substance use in the last month were considered as current users.

### Data quality control issues

The training was given to the data collectors and supervisors on the data collection tool and sampling techniques by the researcher. Supervision was held regularly during the data collection period both by the researcher, co-investigators and supervisors to check on a daily basis for completeness and consistency.

### Data processing and analysis

After cleaning, data was entered, into EPI info version 3.14 then it was exported to SPSS versions 20 for analysis. Descriptive statistics (frequencies and percentages) was used to explain the study participant in relation to study variables. Bivariate and multivariate analysis was used to determine the presences of statistically significant associations between the independent variables and depression and anxiety. Those variables having a p-value less than 0.2 were entered into the multivariate logistic regression model to identify the effect of each independent variable with the outcome variables. The strength of the association was presented by odds ratio and 95% confidence interval. A p-value of < 0.05 on multivariate analyses was considered as statistically significant.

### Ethical considerations

Ethical clearance was obtained from the IRB ethical review board of SPHMMC. After thoroughly discussing, the ultimate purpose and method of the study, written consent was sought from SPHMMC and informed verbal consent was obtained from each respondent. The respondents were informed that their inclusion in the study was voluntary and they were free to withdraw from the study if they are not willing to participate. If any question they do not want to answer they had the right to do so. To ensure the confidentiality of respondents, their names were left out on the questionnaire. All interviews were individually to keep confidentiality. All study participants who become case were linked to an outpatient psychiatric clinic at SPHMMC after getting permission from them.

## Result

### Socio-demographic characteristics of the respondents

A total of 273 participants were included in the study which makes the response rate 98.5%. The mean age of the respondents was 1.61 (SD = 0.65) years. Among the respondents, the majority 129 (48%) were in the age range of 18–21 years, 163 (60.6%) were male, 256 (95.2%) were unmarried, 163 (60.6%) were orthodox religion members and 249 (92.6%) had no additional source of income (Table 1).

**Table 1:** Descriptions of Socio demographic characteristics among undergraduate medical students at SPHMMC, Addis Ababa, Ethiopia, 2016/2017

Variables	Frequency	Percent (%)
Age (years)		
18–21	129	48.0
22–24	115	42.8
25 and above	25	9.3
Sex		
Male	163	60.6
Female	106	39.4
Marital status		
Married	13	4.8
Unmarried	256	95.2
Religion		
Orthodox	163	60.6
Muslim	42	15.6
Protestant	54	20.1
Catholic	7	2.6
Others	3	1.1
Source of income		
Yes	20	7.4
No	249	92.6

### Educational, clinical, psychosocial and substance use characteristics of the respondents

The majority of the participants had the interest to study medical health 222 (82.5%). About 189 (70.3%) of them reported that a short vocational status (part-time work). However, most of the participants who had part-time work 163 (60.6%) reported that the length of the vocational period does not have an impact on their education.

Regarding clinical related characteristics, most of the participant had no history of chronic illness (94.4%) as well as a family history of mental illness (90.3%). Nearly half of the participant 129 (48%) had intermediate social support and 29% of the participant had low social support.

More than half of students had no history of stressful life events in last 6 month 144 (53.5%) and 206 (76.6%) of them had no any history of substance (khat, cigarette, and alcohol) use in the last 3 months (Table 2).

**Table 2:** Description of clinical, psychosocial and substance use characteristics among undergraduate medical students at SPHMMC, Addis Ababa, Ethiopia, 2016/2017

Variables	Frequency	Percent (%)
Level of education		
1st year	58	21.6
2nd year	52	19.3
3rd year	46	17.1
4th year	41	15.2
5th year	37	13.8
6th year	35	13.0
Interest of education		
Yes	222	82.5
No	47	17.5
Vocational status		
Short	189	70.3
Medium	76	28.3
Long	4	1.5
Impact of educational vocation		
Yes	163	60.6
No	106	39.4
Diagnosed chronic illness		
Yes	15	5.6
No	254	94.4
Family history of mental illness		
Yes	26	9.7
No	243	90.3
Social support		
Low social support	78	29.0
Medium social support	129	48.0
Good social support	62	23.0
Stressful life events		
No	144	53.5
Yes	125	46.5
Current substance (khat, cigarette and alcohol) use		
Yes	63	23.4
No	206	76.6

### The magnitude of depression and anxiety among medical students

The prevalence of co-morbid depression and anxiety was found to be 21.20% (16.35% to 26.05%) and prevalence of depression and anxiety was 51.3% (45.37% to 57.23%) and (30.1% 24.66% to 35.54%), respectively.

### Factors associated with depression and anxiety among medical students

The multivariable logistic regression revealed that the odds of anxiety was higher among medical students who are female adjusted odds ratios [AORs, 2.56, 95% CI (1.32, 4.95)], first-year educational level [AOR 12.06, 95% CI (2.18, 66.72)], second-year educational level [AOR 8.99, 95% CI (1.67, 48.45)] and those who had poor/low social support [AOR 5.36, 95% CI (2.08, 13.76) (Table 3). Whereas, being in age ranges between 18 and 21 years [AOR 2.42, 95% CI (1.64, 9.22)], first-year educational level [AOR 1.63, 95% CI (1.43, 6.26)], second-year educational level [AOR 1.39, 95% CI (1.17, 5.18)] and who had stressful life events [AOR 1.61, 95% CI (1.14, 2.76)] were significant associated with depression among medical students (Table 4).

**Table 3:** Factors associated with anxiety among undergraduate medical students at SPHMMC, Addis Ababa, Ethiopia, 2016/2017

Explanatory variables	Anxiety		COR, 95% (CI)	AOR, 95% (CI)
	Yes	No		
Sex				
Male	40	123	1	1
Female	41	65	1.94 (1.14, 3.29)*	2.56 (1.32, 4.95)**
Age				
18–21	51	78	1.39 (0.56, 3.46)	0.26 (0.05, 1.22)
22–24	52	93	0.50 (0.19, 1.31)	0.30 (0.08, 1.10)
25 and above	8	17	1	1
Marital status				
Married	1	11	1	1
Single	80	177	4.97 (0.63, 39.17)	3.23 (0.35, 29.63)
Source of income				
Yes	2	18	1	1
No	79	170	0.24 (0.05, 1.06)	1.450 (0.28, 7.85)
Educational level				
1st year	29	29	4.83 (1.75, 13.39)*	12.06 (2.18, 66.72)**
2nd year	22	30	3.54 (1.26, 9.99)*	8.99 (1.67, 48.45)**
3rd year	10	36	1.34 (0.41, 4.13)	3.42 (0.76, 15.33)
4th year	8	33	1.17 (0.36, 3.78)	2.07 (0.42, 10.15)
5th year	6	31	0.94 (0.27, 3.23)	1.90 (0.41, 8.81)
6th year	6	29	1	1
Interest toward medical education				
Yes	63	159	1	1
No	18	29	1.57 (0.81, 3.02)	1.46 (0.76, 3.18)
Vocational period				
Short	65	124	1.573 (0.16, 15.42)	4.00 (0.33, 48.11)
Medium	15	61	0.738 (0.07, 7.60)	2.28 (0.17, 29.86)
Long	1	4	1	1
Impact of vocational period				
Yes	57	106	1.84 (1.05, 3.21)*	1.47 (0.73, 2.93)
No	24	82	1	1
Diagnosed chronic illness				
Yes	6	9	1.59 (0.55, 4.63)	1.02 (0.20, 3.62)
No	75	179	1	1
Family history of mental illness				
Yes	11	15	1.81 (0.79, 4.14)	2.55 (0.89, 7.23)
No	70	173	1	
Social support				
Low/poor	35	43	3.77 (1.71, 8.31)*	5.36 (2.08, 13.76)**
Medium/intermediate	35	94	1.73 (0.81, 3.69)	2.23 (0.98, 5.32)
High/good	11	51	1	1
Stressful life events				
No	35	109	1	1
Yes	46	79	1.81 (1.07, 3.07)*	1.26 (0.67. 2.34)
Substance use				
Yes	18	46	0.88 (0.47, 1.64)	0.85 (0.41, 1.77)
No	63	142	1	1

n = 269

Diagnosed chronic illness = cardiovascular disease, liver diseases, Epilepsy, HIV/AIDS…

* Significant association (p-value < 0.2 in bivariate)

** Significant association (p-value < 0.05 in multivariate analysis) Hosmer and Lemeshow test = 0.317

## Discussion

### Main findings

In this study, the prevalence of depression and anxiety among medical students and their possible association with various variables were assessed. The results from the current survey revealed that a remarkable proportion of medical students had depression, anxiety as well as comorbid depression and anxiety. One in two medical students found to have depression and one in three and one in five of the students reported anxiety and comorbid depression and anxiety, respectively.

**Table 4:** Factors associated with depression among undergraduate medical students at SPHMMC, Addis Ababa, Ethiopia, 2016/2017

Explanatory variables	Depression		COR, 95% (CI)	AOR, 95% (CI)
	Yes	No		
Sex				
Male	81	80	1	1
Female	50	56	1.11 (0.68, 1.81)	0.99 (0.57, 1.75)
Age				
18–21	77	52	2.63 (1.08, 6.41)*	2.42 (1.64, 9.22)**
22–24	52	63	1.47 (0.60, 3.59)	1.90 (0.63, 5.69)
25 and above	9	16	1	1
Marital status				
Married	1	11	1	1
Single	137	120	0.51 (0.15, 1.74)	0.27 (0.58, 0.98)
Source of income				
Yes	8	12	1	1
No	130	119	1.64 (0.65, 4.15)	2.21 (0.76, 6.40)
Educational level				
1st year	38	20	2.85 (1.20, 6.78)*	1.63 (1.43, 6.26)**
2nd year	34	18	2.83 (1.17, 6.87)*	1.39 (1.17, 5.18)**
3rd year	18	28	0.96 (0.39, 2.37)	0.50 (0.16, 1.65)
4th year	16	25	0.96 (0.38, 2.41)	0.52 (0.16, 1.68)
5th year	18	19	1.42 (0.56, 3.62)	1.01 (0.33, 3.04)
6th year	14	21	1	1
Interest toward medical education				
Yes	109	18	1	1
No	29	113	1.67 (0.88, 3.180	1.86 (0.90, 3.82)
Vocational period				
Short	98	91	1.08 (0.15, 7.81)	1.37 (0.15, 12.85)
Medium	38	38	1.00 (0.13, 7.47)	1.48 (0.15, 14.82)
Long	2	2	1	1
Impact of vocational period				
Yes	82	81	0.90 (0.55, 1.48)	0.81 (0.45, 1.47)
No	56	50	1	1
Diagnosed chronic illness				
Yes	10	5	1.97 (0.65, 5.92)	2.36 (0.69, 8.13)
No	128	126	1	1
Family history of mental illness				
Yes	15	11	1.33 (0.59, 3.01)	1.25 (0.50, 3.11)
No	123	120	1	1
Social support				
Low/poor	41	37	1.53 (1.18, 3.01)*	1.32 (0.63, 2.73)
Medium/intermediate	71	58	1.70 (0.92, 3.13)	1.76 (0.90, 3.43)
High/good	26	36	1	1
Stressful life events				
No	64	80	1	1
Yes	74	51	1.81 (1.12, 2.95)*	1.61 (1.14, 2.76)**
Substance use				
Yes	34	30	1.10 (0.62, 1.93)	1.35 (0.73, 2.53)
No	128	101	1	1

n = 269

Diagnosed chronic illness = cardiovascular disease, liver diseases, Epilepsy, HIV/AIDS…

* Significant association (p-value < 0.2 in bivariate)

** Significant association (p-value < 0.05 in multivariate analysis) Hosmer and Lemeshow test = 0.161

### The prevalence and associated factors of anxiety

The magnitude of anxiety among medical students in the current study (30.1%) was in line with other study conducted in Brazil 33.7% [8]. Contrarily the prevalence of anxiety in the current study was higher than the studies conducted in India (9.8%) [10], and Nepal (5%) [20]. Furthermore, the magnitude of anxiety in the current study was lower than the studies conducted in Egypt (73%) [21], Bahrein (51%) [22], and Brazil (37.2%) [22], Ethiopia) (40.9%) [23], Egypt (78.4%) [12], Pakistan (70.7%) [24], India (66.9%) [25], and in (84.5%) [13]. The possible reasons for the observed difference include the instrument used to measure anxiety, the sample size, the course load and the existing socio-cultural differences among the countries.

Regarding associated factors, the multivariate logistic regression analysis in the current study showed that being females were 2.56 times more likely to have anxiety than males. The possible reason might be increased exposure to acute life events, gender-specific roles, and smaller social networks. The current result is smaller than the study conducted in Northeast Brazil [8] but higher than the study conducted in the University of Gondar, Ethiopia [23].

Students who were the first year medical students had 12.06 times more likely to have anxiety as compared with those who were 6-year medical students. This might be due to exposure to a new environment, new friends, apart from family or new teaching–learning process. This is relatively higher than the study conducted previously in Abbottabad (Pakistan) [15]; this might be due to the difference in socio-demographic, culture, teaching environment or curriculum.

In addition, students who were second-year medical students had 8.99 times more likely to have anxiety as compared with those who were a six-year medical student which is high. This might be due to the number of credit hours of the lesson, exposed to new medical words which are difficult to understand and easily hold and taking the exam frequently.

Regarding social support, students who had poor/low social support were 5.36 times more likely to have anxiety than students who had good social support; this might be due to the feeling of loneliness and lack of social interaction. The current result is higher than the study conducted at the University of Gondar, Ethiopia [23].

### The prevalence and factors associated with depression

The study revealed that the prevalence of depression was (51.3% (CI 46.0, 56.9) high. Regarding the prevalence, the current study’s finding was similar to other studies carried out in the different area of India 49.1% [7] and 51.3% [25]. However, the current study result is higher than the studies conducted in Addis Ababa University (Ethiopia) which were 27.7% [26], in University of Gondar (Ethiopia) which was 40.9% [23], in Madinah (Saudi Arabia) which was 28.3% [27], Sangareddy (India) which was 14% [10] and North East Brazil which was 33.7% [8]; but lower than the study conducted in Egypt which was 63.6% [12], in Tabriz (Iran) which was 62.7% [28], in Malaysia which was 64.4% [13], in India which was 58% [5] and in Karachi (Pakistan) which was 70% [24]. The variation might be due to the difference in sample size and data collection tool which was SRQ-20 with 836 participants in the University of Gondar, Ethiopia [23], DASS-21 with 421 participants in Egypt [12], PQ-2 with 60 participants in Madinah, Saudi Arabia [27], BDI with 175 participants in Tabziz, Iran [28], DASS-21 with 194 participants in Malaysia [13], BDI with participant in India [7], SRQ-20 with 172 participants in North east Brazil [8];difference in data collection tool which was CES-D in Addis Ababa University, Ethiopia [26], DASS-21 in Odisha, India [25], PHQ-9 in India [5], DASS-42 Sangareddy, India [10] and AKUADS in Karachi (Pakistan) [24].

The multivariate logistic regression analysis in the current study showed that students who were in the age interval of 18–21 years were 2.42 times more likely to have depression as compared than those who were 25 and above years old. The current result is similar to the study conducted in the International Islamic University of Malaysia [13].

Regarding educational level, those who are the first year and second-year medical students were 1.63 and 1.39 times more likely to have depression respectively as compared to those who were 6-year medical students. The current finding is similar to the study conducted in India [5]. This might be due to lack of social interaction; unfamiliar types exam schedule; lower grade than anticipated; lack of vacation or break [23] or language problem [7].

With respect to stressful life events, students who had faced one or more of stressful life events in the last 6 months were 1.61 times more likely to have depression as compared to those who had not faced stressful life events in the last 6 months. This might be due to the loss of close family or friends; financial crises or family problems. The current result is lower than the study conducted in India [5] and the University of Gondar, Ethiopia [23].

### The strength and limitation of the study

The present study had several strengths: (1) We used adequate sample size and the participants were involved from a well-defined catchment area; (2) We measure our outcome of interest (depression and anxiety) using standard and validated instrument (HADS); (3) we also estimated the prevalence of coexisting anxiety and depression among the study participants.

However, the current study also had some limitations: first, due to the cross sectional nature of the study the association between different factors and anxiety as well as depression may not imply causation; second, the possibility of recall bias because of the retrospective nature of cross-sectional studies: thirdly, not measuring the effects of other comorbid psychiatric disorders may overestimated or underestimate the observed associations.

## Conclusion

The present study revealed a considerably higher prevalence of comorbid anxiety and depression (21.20%), depression (51.3%), and anxiety (30.1%), among medical students. There was strong evidence of an association between anxiety and female sex, first-year educational level, second-year educational level and having poor/low social support. Whereas a significant association was observed between depression and younger age (18–21 years old), first-year educational level, second-year educational level and having one or more stressful life events in the last 6 months. Screening of depression and anxiety among medical students was recommended. Future studies focusing on better ways of preventing and treating depression and anxiety among medical students are warranted.
